# PrEP implementation in the Asia-Pacific region: opportunities, implementation and barriers

**DOI:** 10.7448/IAS.19.7.21119

**Published:** 2016-10-18

**Authors:** Iryna Zablotska, Andrew E Grulich, Nittaya Phanuphak, Tarandeep Anand, Surang Janyam, Midnight Poonkasetwattana, Rachel Baggaley, Frits van Griensven, Ying-Ru Lo

**Affiliations:** 1 The Kirby Institute, UNSW Australia, Sydney, Australia; 2 The Thai Red Cross AIDS and Research Centre, Bangkok, Thailand; 3 Service Workers In Group Foundation, Bangkok, Thailand; 4 Asia Pacific Coalition on Male Sexual Health (APCOM), Bangkok, Switzerland; 5 HIV Department, World Health Organization, Geneva, Switzerland; 6 HIV Netherlands, Australia, Thailand Research Collaboration (HIV-NAT), Thai Red Cross AIDS Research Center, Bangkok, Thailand; 7 World Health Organization, Regional Office for the Western Pacific, Manila, Philippines

**Keywords:** pre-exposure prophylaxis, the Asia-Pacific region, implementation, demonstration studies, PrEP policy, PrEP awareness, PrEP use, MSM

## Abstract

**Introduction:**

HIV epidemics in the Asia-Pacific region are concentrated among men who have sex with men (MSM) and other key populations. Pre-exposure prophylaxis (PrEP) is an effective HIV prevention intervention and could be a potential game changer in the region. We discuss the progress towards PrEP implementation in the Asia-Pacific region, including opportunities and barriers.

**Discussion:**

Awareness about PrEP in the Asia-Pacific is still low and so are its levels of use. A high proportion of MSM who are aware of PrEP are willing to use it. Key PrEP implementation barriers include poor knowledge about PrEP, limited access to PrEP, weak or non-existent HIV prevention programmes for MSM and other key populations, high cost of PrEP, stigma and discrimination against key populations and restrictive laws in some countries. Only several clinical trials, demonstration projects and a few larger-scale implementation studies have been implemented so far in Thailand and Australia. However, novel approaches to PrEP implementation have emerged: researcher-, facility- and community-led models of care, with PrEP services for fee and for free. The WHO consolidated guidelines on HIV testing, treatment and prevention call for an expanded access to PrEP worldwide and have provided guidance on PrEP implementation in the region. Some countries like Australia have released national PrEP guidelines. There are growing community leadership and consultation processes to initiate PrEP implementation in Asia and the Pacific.

**Conclusions:**

Countries of the Asia-Pacific region will benefit from adding PrEP to their HIV prevention packages, but for many this is a critical step that requires resourcing. Having an impact on the HIV epidemic requires investment. The next years should see the region transitioning from limited PrEP implementation projects to growing access to PrEP and expansion of HIV prevention programmes.

## Introduction

In 2014, the Asia-Pacific region was home to more than half of the world's population and 15.2% of the estimated 36.9 million people living with HIV globally [1]. Most of the region's HIV-positive people (~90%) live in five countries – China, India, Indonesia, Thailand and Vietnam [2]. Epidemics in the region can be characterized as concentrated and growing in key populations, mainly among men who have sex with men (MSM), particularly young MSM, or shifting towards MSM as the main mode of transmission. Below, we focus on 12 countries with the largest number of people living with HIV (see Table 1) [3].

**Table 1:** HIV epidemics in 12 countries of Asia-Pacific region (end of 2014)

#	Country	Estimated number of new infections in 2014^a^	HIV prevalence in 2014, adults 15 to 49^a^	Key characteristics of the epidemic	Levels of PrEP awareness, acceptability and use	PrEP demonstration and implementation projects
1	Australia	1300 (630 to 1700)	0.2%	Sexual transmission, mainly among MSM (70% of new cases in 2014).	In 2013, 77% of HIV non-positive MSM had heard of PrEP; 29% knew someone taking PrEP; 17% discussed PrEP with a doctor [25] In 2011, 2.5% of HIV non-positive MSM who engaged in condomless anal intercourse reported ever using PrEP informally [25]; levels of PrEP use have remained low since then. [26,27]	1. **Demonstration project PRELDUE [34] (2014 to present)**: 300 participants taking daily oral TDF/FTC in Sydney, Australia. **Aim**: Establish and evaluate PrEP service delivery model in NSW, Australia; evaluate PrEP acceptability, adherence, behaviour, STI incidence among MSM. **Sponsor**: The Kirby Institute, UNSW Australia. 2. **Implementation study EPIC-NSW [30] (2016 to 2018**): 3700 participants taking daily oral TDF/FTC in NSW, Australia. **Aim**: Evaluate effect on HIV incidence from providing PrEP to high-risk population group/s. **Sponsor**: The Kirby Institute, UNSW Australia. 3. **Demonstration project VicPrEP [35] (2014 to 2019**): 115 MSM and women at high risk of HIV infection taking daily oral TDF/FTC in Melbourne, Australia. **Aim**: Determine the effectiveness of PrEP in the local setting and the factors contributing to its success. **Sponsor**: Alfred Health, in conjunction with the Victorian AIDS Council. 4. **Demonstration project QPrEP [36] (2016 to present):** 50 MSM taking daily oral TDF/FTC in Cairns, Australia. **Aim**: Evaluate PrEP delivery in Queensland health services. **Sponsor**: Queensland Health.
2	Cambodia	860 (440 to 2200)	0.6%	Sexual transmission; an urban epidemic among three key populations and their partners.	N/A^b^	–
3	China	60,000 (15,000 to 130,000)	0.1%	Sexual transmission, mainly among MSM; nationally declining epidemic, with regional variations.	In 2009 to 2010, 11.2% of MSM in Beijing had heard of PrEP; 67.8% were willing to accept it if it was effective [19]. In 2009 to 2010, 22% of MSM in South-Western China had heard of PrEP; <1% had ever used it; 64% were willing to take PrEP after having it explained to them [19].	–
4	India^c^	86,000 (56,000 to 129,000)	0.3%	HIV epidemic heterogeneous in its distribution, concentrated among high-risk groups. Nationally declining epidemic, with regional variations.	In 2010 to 2011, >75% of MSM reported they would be willing to take PrEP, mainly in an injectable form [20]. In 2012, MSM participating in a qualitative study in Chennai had not heard about PrEP previously, but once it was explained, were interested and likely to use it as an alternative to condoms [22]. In 2013, a qualitative review of PrEP acceptability research found scarce research on oral PrEP in India, generally limited acceptability of oral PrEP among high-risk groups (both men and women) [21].	1. **Prospective intervention PrEP-India (April 2016–June 2018, not active yet):** 2000 SW taking PrEP plus peer educator home visits every other day, as part of regular DMSC (Kolkata) and Ashodaya (Mysore/Mandya) outreach prevention activities. **Aim**: Demonstrate safety and effective delivery of daily oral PrEP as part of an HIV combination preventive intervention for sex workers in Kolkata and Mysore-Mandya, India. **Sponsor**: University of Manitoba.
5	Indonesia	69,000 (63,000 to 76,000)	0.5%	Shift from injecting drug use to sexual transmission. Key population groups involved in the epidemic: transgender SW, male and female SW, PWID and MSM.	N/A	–
6	Malaysia	6200 (5700 to 6800)	0.4%	In earlier phases epidemic was driven by PWID, then shifted to sexual transmission, with heterosexual/homosexual ratio=2:1.	N/A	–
7	Myanmar	8700 (7800 to 9500)	0.7%	Epidemic mainly evolving in PWID, but increasing in this group and in MSM.	N/A	–
8	Nepal	1500 (1300 to 1600)	0.2%	Most HIV infections happen in low-risk males and females, PWID, MSM and TG. Infections have declined in PWID but have increased in MSM.	N/A	–
9	Papua New Guinea	2000 (1500 to 2500)	0.7%	Epidemic recently downgraded from generalized to concentrated; men and women who sell sex and MSM are key population groups engaged in the epidemic.	N/A	–
10	Philippines	6400 (1500 to 12,000)	0.1%	Homosexual contact is the main mode of HIV transmission, and people who inject drugs in certain geographical areas.	N/A	–
11	Thailand	7900 (3700 to 13,000	1.1%	Key affected populations: FSW, MSW, MSM and TG with very high incidence among young MSM. Number of new HIV infections continues to decline but at a slower pace since 2010.	In 2012, 66% of MSM and TG survey respondents in Northern Thailand were aware of PrEP; 41% of MSM and 37% of TG were “very likely” to use PrEP [37].	1. **PrEP implementation project in MSM and TG (2015 to 2018):** 600 MSM and TG, including 300 in community-based settings (four CBOs: SWING Foundation in Bangkok and Pattaya, Rainbow Sky Association of Thailand in Bangkok and Sisters Foundation in Pattaya) and 300 in the facility-based setting (two hospitals in the Test and Treat project in Bangkok and Pathumthani), taking daily oral TDF/FTC. **Aim**: Assess PrEP uptake and factors influencing decision-making among MSM and TG. **Sponsor**: Department of Disease Control, Thai MOPH, and Thailand MOPH-US CDC Collaboration, Thai Red Cross AIDS Research Centre. 2. **Observational study Princess PrEP project (2016 to 2018):** 3000 MSM and TG taking daily oral TDF/FTC. **Aim**: Expand oral PrEP access among MSM and TG through seven CBOs in Bangkok, Pattaya, Chiang Mai and Hat Yai; study the characteristics of these MSM and TG. **Sponsor**: Thai Red Cross AIDS Research Centre, USAID RDMA, FHI360.
						3. **Observational study PrEP-30 project [38] (2014 to present) [38]:** Unlimited number of MSM, TG, women taking daily oral TDF/FTC. **Aim:** Assess feasibility of a self-paid oral PrEP programme (clients pay 30 THB per day or 1 USD per day to cover the cost of generic TDF/FTC, laboratory screening, monitoring and counselling) at the Thai Red Cross Anonymous Clinic. **Sponsor**: Thai Red Cross AIDS Research Centre. 4. **Open label intervention COPE4YMSM (2015 to 2020):** 1240 young MSM, including 620 who chose PrEP and 620 who did not choose PrEP. Combination intervention: daily oral TDF/FTC with or without regular HIV testing, risk reduction counselling, condom and condom-compatible lubricant distribution. **Aim**: Assess the effectiveness of a combination HIV prevention intervention with and without daily oral Truvada^®^ (PrEP). **Sponsor**: Johns Hopkins Bloomberg School of Public Health. 5. **Implementation study PrEP @ PIMAN (2015 to 2017)**: 200 MSM and TG women in Chiang Mai. HIV testing, counselling and daily oral PrEP. **Aim**: Assess acceptability of daily oral PrEP among MSM and TGW, factors that influence the decision to take daily oral PrEP and assess adherence to PrEP among PrEP users. **Sponsor**: Research Institute for Health Sciences, Chiang Mai University, Chiang Mai.
12	Vietnam	15,000 (13,000 to 16,000)	0.5%	Number of new infections rapidly declined between 2007 and 2009 and stabilized after that. Transmission stabilized and declined in all groups except MSM. Infections are increasing in remote and mountainous areas.	N/A	–

FSW, female sex worker; MSM, men who have sex with men; MSW, male sex worker; PrEP, pre-exposure prophylaxis; PWID, person who injects drugs; STI, sexually transmitted infection; SW, sex workers; TGW, transgender women; CBO, community-based organization.

a Data about new HIV infections and HIV prevalence provided by WHO.

b Data not available.

c India HIV Estimate 2015, NACO 2015.

From 2000 to 2014, the estimated number of new infections in the Asia-Pacific declined by 31%, although it stalled during the last eight years, and the estimated number of AIDS-related deaths increased by 11% [1]. Globally, HIV incidence decreased by 35% after 2000 and AIDS-related deaths dropped by 42% after 2004 [4]. New HIV infections declined in some countries in the region (India, Myanmar, Thailand, Cambodia and Vietnam) but increased in others (Pakistan, Philippines and Indonesia).

HIV prevalence is 5 to 15 times higher among MSM compared to the general population in South and South-East Asia [5]. Infections among female sex workers (FSW) have slowed but remain important contributors to HIV transmission in the region [6]. Limited data are available about the HIV epidemics in transgender people (TG), estimated to number 9 to 9.5 million in the region [7], and small-scale research is mostly limited to TG women who have sex with men. In several cities HIV prevalence in this group was substantially higher than in the general population of reproductive age and even higher than in MSM [8].

While disproportionately affected by HIV, the key risk populations are mostly underserved by HIV prevention programmes. Throughout Asia, less than 60% of MSM and FSW know where to get tested for HIV or have received condoms through distribution programmes [9] (level of condom use >80% is considered to have an impact on an HIV epidemic [10]). Condom promotion programmes are not reaching men at a sufficiently high level: rates of condom use at last sex among MSM are half of the rate in FSW (two-thirds of that among male sex workers (MSW)) [11]. Distribution of needles and syringes is less than half of the recommended 200 per year per person who injects drugs (PWID) [9], and safe sex messages for PWID are mostly absent [11].

Studies in MSM have provided evidence of the safety and efficacy of daily tenofovir disoproxil fumarate (TDF), alone or in combination with emtricitabine (TDF/FTC) for HIV pre-exposure prophylaxis (PrEP) [12–15]. Oral PrEP is also effective for HIV prevention in women [9]. A relatively modest level of PrEP efficacy was established for PWID [16]. No studies were conducted to evaluate PrEP efficacy specifically in TG people, but TG participants of the iPrEx trial were protected from HIV if they had taken PrEP [17].

PrEP implementation may be more feasible and cost-effective in settings with concentrated HIV epidemics and defined population groups who may benefit from it [18]. It also appears to be particularly effective in MSM, and many of the region's HIV epidemics are concentrated in MSM. In this paper, we discuss the progress towards PrEP implementation in the Asia-Pacific region, including opportunities and barriers.

## Discussion

### Levels of PrEP awareness and acceptability

Recently, several surveys on PrEP awareness and use have been reported from the Asia-Pacific, including from China [19], India [20–22], Thailand [23] and Australia [24–27] (see Table 1). Overall, there has been no systematic data collection about PrEP in the region. Where available, results from published studies are not consistent and are incomparable, as different measures were used. Overall awareness about PrEP among Asian MSM is very low outside of Australia and Thailand, but among those aware about PrEP, willingness to use it is relatively high. Almost no PrEP-related data exist about other population groups. The Asia and Pacific Coalition on Male Sexual Health (APCOM) is planning surveys of PrEP acceptability among MSM and their health providers in countries with high HIV prevalence to inform future PrEP implementation [28].

### PrEP trials and demonstration studies

Some sites in Asia have participated in randomized trials of PrEP, which helped to generate local evidence and advocacy for further PrEP research and implementation. Among MSM, Chiang Mai in Thailand was a site of the original iPrEx study [13] and the Silom Community Clinic in Bangkok participates in the ongoing HPTN 067/ADAPT study examining alternative PrEP dosing in MSM and TG women [29]. The only study to evaluate PrEP efficacy in PWID globally was conducted in Bangkok [16]. There have been no randomized clinical trials of PrEP focused on heterosexuals or TG women in the region.

PrEP research in the region is moving to PrEP demonstration studies (see Table 1). They are a critical step to implementation, because they generate experiences and highlight local barriers to PrEP uptake and challenges for future PrEP implementation (e.g. adherence to PrEP) in the region.

In Australia, there are currently three demonstration projects underway and a large-scale implementation trial, EPIC-NSW (Expanded PrEP Implementation in Communities in New South Wales) [30]. Based in the state with the largest overall population and MSM community [31], EPIC-NSW is a partnership of the key NSW government, community, medical and research organizations. The project aims to rapidly scale up PrEP implementation to about 3700 high-risk participants [32]. Its scope and size were estimated based on the high-risk PrEP-eligibility risk criteria of the NSW PrEP guidelines, data from previous Australian studies about HIV transmission factors in the Australian context and on estimation of the expected impact this project may have on the HIV epidemic in NSW [33].

Thailand, the first country in the region to host the PrEP trial sites, has several PrEP implementation projects underway (see Table 1). The PrEP-30 project [38], led by the Thai Red Cross AIDS Research Centre (TRCARC), is using a fee-based PrEP service delivery model. The PrEP implementation project for MSM and TG evaluates a community-led PrEP service delivery model (services provided by non-medical staff of the four community-based organizations) and a facility-based model (services provided by two hospitals). The Princess PrEP project (also based on the community-led PrEP service delivery model) is the result of the TRCARC's Princess Soamsawali Prevention of Mother-to-Child Transmission Fund, which recently transformed into the Princess Soamsawali Fund for HIV Prevention. It aims to provide PrEP to 3000 MSM and TG women over three years, starting in 2016. TRCARC has also started exploring innovative technology-based interventions [39], especially for targeting young and closeted individuals and for those who are typically hard to reach. Adam's Love has been the leading online outreach platform for PrEP awareness and counselling among Thai MSM and TG to facilitate recruitment and enrolment into the PrEP-30 and Princess PrEP Projects [39]. Its online-to-offline recruitment model has demonstrated the feasibility of reaching the previously unreached MSM and TG through the use of technology-based interventions, linking them to offline PrEP services, helping them with adherence and following them up through the use of its innovative electronic health record system.

PrEP pilot projects are being planned in other Asian settings including Manila, the Philippines, and Ho Chi Minh City, Vietnam.

### PrEP implementation issues

Almost four years after PrEP approval by the US Food and Drug Administration [40], PrEP implementation in the United States remains a work in progress: the number of people receiving PrEP started to increase rapidly in about 2014 [41]. Elsewhere, PrEP implementation has been limited due to a number of issues.

Although awareness about PrEP has been increasing [23], its levels and pace of growth are still insufficient to produce a reasonable service demand. Scale-up of education about PrEP is necessary in the communities around the region.

Access to PrEP is part of a much broader issue in the Asia-Pacific. Many countries are struggling to provide access to antiretroviral therapy (ART) to all eligible people with HIV. In 2014, 36% of all adults living with HIV in the Asia-Pacific were receiving treatment, compared to 40% globally [42]. Some countries have made significant progress: for example, 83% of eligible HIV-positive people in Thailand and 82% in Cambodia were on ART in 2012. However, some countries with large numbers of eligible people (under the 2010 WHO HIV treatment guidelines) were lagging behind with respect to ART coverage (examples include India (57%), Indonesia (18%) and Myanmar (45%)).

HIV testing is the entry point for HIV prevention and treatment, but the regional median coverage of MSM with testing remains low (less than 50% of MSM in the majority of countries with available data in 2013 [43]). The lack of community-based testing and policies to allow lay and peer provider testing are barriers to reaching MSM though community outreach approaches in many countries. HIV self-testing has the potential to reach people who do not have contact with health services. It is available informally via the Internet and pharmacies but is not as yet included in national testing policies, and most countries do not have quality assurance systems in place to ensure the control of and access to high-quality testing services [44].

In some countries, such as Myanmar [45], Cambodia [46] and the Philippines [47], old punitive laws are still in action. As a result, stigma and discrimination against marginalized groups remain common barriers to PrEP implementation. Of note, most countries acknowledge the existent issues and some have achieved progress in eliminating stigma and discrimination. For example, the Vietnam Health Insurance Law is intended to cover 100% of the costs of HIV-related services for poor people and 95% for the near-poor [48].

The cost of antiretrovirals (ARVs) has been a major problem. In many Asian countries, ARVs are not available outside of government-accredited treatment hubs (e.g. Philippines) [42] due to high medication cost. In some countries (e.g. Thailand, Vietnam, Singapore, Malaysia), ARV medications are available in the private sector. Issues that impact access to PrEP are related to drug licensing (e.g. generic TDF/FTC is not yet approved by regulatory authorities in Australia) and pricing. Although generic TDF/FTC is produced in the region, it is not registered for preventive use there. Furthermore, restrictions on access to ARVs due to the complexity of intellectual property laws may affect the rapid implementation of PrEP in many countries in the region [49]. Meanwhile, projects like PrEP-30 in Thailand have introduced a fee-based PrEP service delivery model with a user fee of 30 Thai baht (approximately US$1) per day to cover facility and physician fees, laboratory testing and generic TDF/FTC costs [38]. In Australia, TDF/FTC (marketed as Truvada^®^ by Gilead Sciences, Inc.) was approved for preventive use in May 2016. However, to make it affordable to all Australians, the medications should also be approved under the national Pharmaceutical Benefits Scheme. Several innovative approaches have emerged to start PrEP implementation, including investment by state governments in large-scale PrEP implementation studies in NSW [50] and Victoria [51], clinicians prescribing PrEP outside demonstration projects and community initiatives providing education about access to PrEP [52].

Even if PrEP was made available, there is little practical guidance for countries with concentrated epidemics on how to target PrEP to high-risk groups. WHO guidelines recommend targeting PrEP to population groups with HIV sero-incidence above 3 per 100 person-years [53]. HIV infection in the Asia-Pacific region is concentrated among MSM and other key populations, but there is a general lack of data about HIV sero-incidence in these groups. In Australia, eligibility criteria for PrEP target high-risk MSM based on local HIV sero-incidence data derived from research cohorts [33].

There is another reason for a lack of PrEP uptake – simply, provision of ARVs for HIV-negative people as primary HIV prophylaxis is a new service just about everywhere around the world. Different service delivery models have recently emerged across the Asia-Pacific region: large-scale implementation trials led by researchers [15,50], as well as facility-based and community-based initiatives (e.g. in Thailand). In Thailand and Australia, some projects are investigating the use of PrEP navigators and nurses in providing PrEP services.

The WHO consolidated guidelines on HIV testing, treatment and prevention call for an expanded access to PrEP [54]. No nations in the Asian region provide free PrEP to those at risk of HIV, although there are scale-up implementation projects in Thailand and Australia that provide free PrEP to a limited number of participants. Some PrEP access projects, like the PrEP-30 project in Bangkok, charge nominal fees for PrEP and associated services [38]. However, even in high-income countries, such PrEP access programmes are only a temporary solution. Thus, solutions are important for Truvada costing and widespread access to generic TDF/FTC.

### Policy on PrEP

In 2012, the WHO published a framework for PrEP implementation and research [55] and consolidated guidance on HIV prevention, diagnosis, treatment and care for key populations internationally [54]. Some countries have chosen to develop their own prescriber guidelines. Australia released NSW state and then national guidance for PrEP prescribers in 2014 to 2015 [33,56]. Australian guidance differs from current clinical guidelines in the United States [56,57], South Africa [58] and the European Union [59] in its approach to determining eligibility for PrEP. In the current environment with limited access to TDF/FTC, the guidelines target daily PrEP to those at high and ongoing risk of HIV infection, which in the Australian context is mainly MSM [56]. These recommendations may expand to those at only moderate risk of HIV as access to PrEP improves.

Other countries in the region have also been working on PrEP policy development. Thailand included a PrEP recommendation for high-risk populations in the Thailand National Guidelines on HIV/AIDS Treatment and Prevention in 2014 [60]. The new Malaysian *National Strategic Plan for Ending AIDS 2016-2030* also recommends studying uptake of self-paid PrEP [61].

### Community involvement and leadership

The Asia-Pacific hosts some prominent examples of community participation, leadership and partnership in promoting PrEP implementation. APCOM is a regional and global advocate of delivering PrEP to MSM. In 2015, APCOM hosted the first community-led regional dialogue in Bangkok – a community and stakeholder consultation, to inform the development of PrEP demonstration projects among MSM in countries with high HIV prevalence where PrEP rollout is in planning [62].

Examples of community leadership as to PrEP have emerged mainly in two countries. In Australia, traditionally active partners in HIV response [63], the AIDS Councils of NSW and Victoria, are also key partners in rolling out PrEP demonstration projects. The state Ending HIV campaigns promoted by these AIDS Councils are now supporting the implementation of PrEP [64]. In 2015, new examples of community advocacy for PrEP have also emerged, mostly driven by limited access to and high cost of PrEP, such as community information resources on how to access PrEP outside demonstration projects in Australia (the PrEP Access Options paper [15]). In Thailand, the SWING Foundation, Rainbow Sky Association of Thailand and Sisters Foundation have worked closely with TRCARC under USAID/PEPFAR on generating PrEP awareness and demand and providing PrEP services.

However, even more community involvement is needed to advocate for the need for PrEP in other countries and in other key population groups yet overlooked for PrEP implementation (particularly TG people, FSW, MSW and their clients).

## Conclusions

Issues specific to HIV epidemics in Asia-Pacific, particularly the concentration of HIV among MSM and other key population groups, make PrEP a suitable HIV prevention intervention for the region. Very few countries in the Asia-Pacific region have implemented PrEP, and those that have are in the inception stages of PrEP implementation. Overall there is a general lack of available data about progress towards PrEP implementation in counties other than Thailand and Australia. There have been clinical trials and demonstration projects of PrEP in Thailand and Australia, and several more are in planning across the region. However, in countries other than Australia and Thailand, there is little information about government policy and strategy, as well as demand from communities about PrEP implementation.

Awareness about PrEP in the region is growing. Among MSM who have heard about PrEP, in some settings there are high levels of acceptability and willingness to use PrEP but low levels of uptake, while less is known about other population groups. In most countries in the region, there is no systematic collection of information about PrEP awareness, acceptability and use in key population groups to understand PrEP needs and inform introduction of PrEP in national HIV strategies. Few data are available about PrEP use among MSM, and hardly any data are available in other key population groups, which have been generally overlooked in PrEP access programmes. Issues of stigma and discrimination are common in many counties. Some still have restrictive laws that have implications for access by the key risk populations to HIV prevention in general and to PrEP. Moreover, access to ART for eligible people living with HIV has been improving but not to the level expected yet, so introduction of PrEP is challenging in many countries of the region and costly for all.

Countries of the Asia-Pacific will benefit from adding PrEP to their HIV prevention packages, but for many this is a critical step that requires resources and access to TDF/FTC. Having an impact on the HIV epidemic requires investment in PrEP. The interest and leadership in the region associated with bringing this intervention to communities is encouraging and exciting. There are already prominent examples of action, at least in Thailand and Australia. The next years should see the region transitioning from limited PrEP implementation projects to increasing access to PrEP and expansion of HIV prevention programmes.
